# Sense of belonging to local community in small-to-medium sized Canadian urban areas: a comparison of immigrant and Canadian-born residents

**DOI:** 10.1186/s40359-015-0085-0

**Published:** 2015-08-20

**Authors:** Peter Kitchen, Allison M. Williams, Melissa Gallina

**Affiliations:** McMaster Institute of Environment & Health, McMaster University, 1280 Main Street West, Hamilton, ON Canada; School of Geography and Earth Sciences, McMaster University, Hamilton, ON Canada

## Abstract

**Background:**

Sense of belonging is recognized as an important determinant of psychological and physical well-being. Research in Canada has shown that sense of belonging has increased in recent years although important variations exist between regions and among certain ethnic groups.

**Methods:**

The objective of this paper is to examine differences in sense of belonging to local community between Canadian-born and immigrant residents in three small-to-medium sized urban areas using primary data collected in: 1) Charlottetown, PEI; 2) Hamilton, Ontario, and 3) Saskatoon, Saskatchewan. A mixed method approach is used in the analysis. First, a household telephone survey (*n* = 1529) asked respondents to rate their sense of belonging. This data was analyzed by way of summary statistics and ordered logistic regression. Second, a series of focus groups with immigrants in the three cities included questions on belonging and well-being (*n* = 11).

**Results:**

The research found that sense of belonging is very high in the overall sample and in the three study sites, particularly in Charlottetown, and that there are no significant differences in levels of belonging between Canadian-born and immigrant respondents. However, among immigrants, sense of belonging was significantly lower for those living in Canada for 5 years or less. Consistent with the literature, positive mental health was found to be strongly associated with a positive sense of belonging for both Canadian-born and immigrant respondents. For immigrants, positive sense of belonging was associated with full-time work and home-ownership, two factors not associated with the Canadian-born population. The paper also revealed that immigrants placed greater importance on knowing their neighbours on a first name basis and generally trusting people as determinants of a positive sense of belonging. Finally, the focus groups revealed that in addition to displaying a sense of belonging to their city of residence, immigrants also maintain strong feelings of belonging to their ethnic group.

**Conclusions:**

The paper concludes by offering several public health recommendations on how belonging can be enhanced among recent immigrants in smaller Canadian cities; these include improved coordination of services in order to contribute to a less overwhelming settlement process for immigrants.

## Background

### Introduction

The objective of this paper is to assess how sense of belonging differs among residents of small-to-medium sized Canadian urban areas according to immigrant status. Canada has the highest level of immigration among all G-8 countries and one of the highest in the developed world. The majority of newcomers settle in Canada’s largest cities, particularly Toronto, Montreal and Vancouver. Correspondingly, the bulk of research on immigrant settlement patterns and issues concentrates on these large urban centres. At the same time, immigration is an important feature of smaller urban areas in Canada, being often the primary source of population growth and, to a certain extent, economic development. However, relatively little is known about the quality of life of immigrants in these smaller urban places, and how it differs from the Canadian-born population residing there. An important aspect of quality of life is the sense of belonging residents feel to the local community (Costanza et al. 2007). The literature on this topic points to clear links between sense of belonging and a person’s health and social well-being (Ross 2002; Choenarom et al. 2005; Shields 2008; Kitchen et al. 2012a). It is well established that immigrants to Canada face important challenges, including finding meaningful employment commensurate to their qualifications, and integrating into Canadian society. Even less is known about immigrants’ sense of belonging.

This paper aims to address these multiple issues by comparing the Canadian-born population to immigrants living in three small-to-medium sized Canadian urban areas: 1) Charlottetown, Prince Edward Island; 2) Hamilton, Ontario, and; 3) Saskatoon, Saskatchewan. The following contextual information for each of these three sites was obtained from Statistics Canada’s 2011 Census (Statistics 2013a) and the 2011 National Household Survey (Statistics 2013b) and Citizenship and Immigration Canada (Citizenship and Immigration Canada (CIC) 2011) (Table 1). In 2011, the City of Charlottetown had a population of 34,562. Between 2006 and 2011, it experienced a population growth of 7.4 %. The immigrant population in 2011 was 3435 (about 10 % of the total). The top source countries were China, Iran, the United Kingdom and the United States. Recent immigrants (those arriving between 2006 and 2011) totalled 1850 in 2011 with the top source countries being China (960), Iran (270), the UK (45) and Turkey (45). In 2011, the City of Hamilton had a population of 519,949 and witnessed a population growth of 3.1 % between 2006 and 2011. The immigrant population was 125,010 (about 24 % of the total). The top source countries were the UK, Italy, Poland and India. Recent immigrants totalled 14,820 in 2011 with the top source countries being the Philippines (1275), Iraq (1120), India (895) and the US (860). In 2011, the City of Saskatoon had a population of 222,189 and experienced a population growth of 10 % during the 5 preceding years. The immigrant population was 26,050 (about 12 % of the total). The top source countries were the Philippines, China, the UK and Pakistan. In 2011, recent immigrants totalled 11,190 with the top sources countries being the Philippines (4155), China (900), India (605) and Pakistan (450). Table 1 (Citizenship and Immigration Canada (CIC) 2011) shows levels of immigration (Permanent Residents) for selected sites in Canada between 2009 and 2013, including the three study areas. It illustrates that immigrant numbers have declined over time in both Charlottetown and Hamilton, and have remained relatively stable in Saskatoon.

**Table 1:** Permanent residents by selected urban areas and provinces (% of provincial total for urban areas in parentheses)

	2009	2010	2011	2012	2013
Charlottetown	1,630 (95 %)	2,493 (97 %)	1,665 (96 %)	982 (90 %)	851 (85 %)
PEI total	1,723	2,581	1,731	1,088	998
Hamilton	3,778 (4 %)	4,003 (3 %)	3,296 (3 %)	4,075 (4 %)	3,213 (3 %)
Toronto	82,639 (77 %)	92,182 (78 %)	77,759 (78 %)	77,399 (78 %)	81,691 (79 %)
Ottawa-Gatineau	6,297 (6 %)	7,172 (6 %)	6,411 (6 %)	6,085 (6 %)	5,978 (6 %)
Ontario total	106,861	118,111	99,458	99,154	103,494
Saskatoon	2,564 (37 %)	3,176 (42 %)	3,796 (42 %)	4,455 (40 %)	3,739 (35 %)
Regina	2,058 (30 %)	2,567 (34 %)	3,202 (36 %)	3,932 (35 %)	3,654 (34 %)
Saskatchewan total	6,890	7,615	8,955	11,177	10,679
Canada total	252,174	280,691	248,748	257,887	258,953

Source: Citizenship and Immigration Canada (2013)

The research employs a mixed method design, with data being first collected from a household quality of life survey conducted via telephone in the three cities during the spring and summer of 2012. A total of 1529 respondents completed the telephone survey, of which 413 (27 %) were immigrants. One of the survey questions inquired about the respondents’ sense of belonging. Several other questions asked about health, sense of place and community conditions. Following a preliminary analysis of the survey data, a total of eleven focus groups with immigrants were held in the three cities. The results associated with the telephone survey provide useful insights into the quality of life of a segment of the Canadian population (namely immigrants in smaller cities) that has often been overlooked by research focused primarily on the country’s largest urban areas.

### Literature review

Sense of belonging is a concept related to quality of life, encompassing a feeling that individuals matter to one another and to a group. Maslow (Maslow 1954) suggested that sense of belonging is a basic human need. Hagerty et al. (Hagerty et al. 1992) define sense of belonging as “the experience of personal involvement in a system or environment so that persons feel themselves to be an integral part of that system or environment” (173). Systems encompass both relationship and organizations; environments can be natural or cultural. Furthermore, sense of belonging is composed of two dimensions: (1) ‘valued involvement’, which includes feeling valued, accepted, and needed; and, (2) ‘fit’, an individual’s perception that they complement the system or environment (Ross 2002). According to the Canadian Community Health Survey (CCHS), the proportion of Canadians reporting a strong or somewhat strong sense of belonging has increased in recent years; in 2000/2001 it was 56 % of Canadians and in 2007/2008 it had risen to 68 % (Ross 2002; Shields 2008; Kitchen et al. 2012a).

Sense of belonging is recognized as an important determinant of psychological and physical well-being (Hagerty & Patusky 1995). Consequently, greater feelings of belonging have been associated with better social and psychological functioning (Hagerty et al. 1996). Sense of belonging influences mental health; low levels of sense of belonging have been associated with higher rates of depression (Choenarom et al. 2005; Hagerty & Williams 1999). In each cycle of the CCHS mentioned above, perceptions of physical and mental health were strongly related to feelings of community belonging (Citizenship and Immigration Canada (CIC) 2011; Maslow 1954; Hagerty et al. 1992). Engendering sense of belonging is clearly a public health issue given its relationship with physical and psychological well-being.

As demonstrated by the 2005 CCHS, evaluations of community belonging vary according to home language and cultural group (Maslow 1954). In comparison to those who speak English in the home (68 %), those speaking other languages were less likely to report strong/somewhat strong feelings of belonging (60 %) (Maslow 1954). Similarly, whites were more likely to express strong/somewhat strong feelings of belonging (65 %) compared to a number of other cultural groups: Koreans (50 %); Chinese (52 %); Southeast Asians (52 %), and; Latin Americans (54 %) (Maslow 1954). However, South Asians (74 %) were more likely to express strong/ somewhat strong feelings of belonging (Maslow 1954). These numbers reflect the fact that newcomers to Canada tend to have weaker feelings of belonging; this is especially true for visible minorities (Erickson 2007; Soroka et al. 2007). It is important to point out, however, that the categories employed in the CCHS are quite broad and that considerable diversity likely exists within each in terms of race, ethnicity and nationality. Despite recent improvements, racism and xenophobia remain substantial issues within Canadian society (Fontana 2003). Experiences of discrimination/intolerance, as well as the loss of relationships during immigration, undermine feelings of belonging (Sonn 2002; Reitz & Banerjee 2007), although sense of belonging tends to increase with length of residency in Canada (Hagerty et al. 1992). Even so, however, analysis of the 2007/2008 CCHS did not find a significant difference in evaluations of sense of belonging according to immigrant status (Hagerty et al. 1992).

According to Hagerty and Patusky (Maslow 1954), sense of belonging is closely related to social integration. Frideres (Frideres 2008) defines social integration as “the process by which newcomers become part of the social, cultural and institutional fabric of the host community or society while at the same time retaining their own cultural identity” (80). The level of social integration depends on whether these social contacts and group memberships occur within an ethnic group, the host population or a combination of both (Fontana 2003). Social integration improves as the quantity and quality of relationships with the host population increases (Fontana 2003).

Antonsich (Antonsich 2010) conducted an extensive cross-discipline review of the concept of belonging and argues that in an era of transnational migration, it is ‘back on the agenda’ (652). He asserts that on the one hand, belonging is central to issues of social cohesion, loyalty, commitment and ‘we’ feelings but on the other, is questioned in its “territorialized dimension or in its fixed stable boundaries” (652). The author observes that an open question is whether the growing ethnic and cultural diversity of modern societies can result in the creation of communities of belonging beyond communities of identity. From a geography perspective, Gilmartin (Gilmartin 2008) observes that new approaches to the study of migration, incorporating qualitative techniques, have provided theoretical insight into questions of identity and belonging. These include the concepts of “transnationalism and translocalism, and…scales of belonging that range from citizenship to the home” (1837).

Building on the conceptual work of Antonsich (Antonsich 2010), Gilmartin (Gilmartin 2008) and others, Huot et al. (Huot et al. 2014) explored the sense of belonging of French speaking visible minority immigrants in London, Ontario in the context of official bilingualism and official multiculturalism. Over a 10-month period a series of interviews were conducted with eight study participants complimented by the creation of mental maps, which served to identify the places participants’ regularly visited and to discuss what they did at these places. The authors found that each of the participants experienced significant challenges in their attempt to integrate into the host society, which in turn influenced the ways they negotiated belonging. These challenges included discrimination, racism and diminished expectations that went along with their linguistic skills. As Huot et al (Huot et al. 2014) observe, “the participants’ development of a personal sense of place-belongingness over time was influenced by the politics of belonging occurring within the socio-geographic contexts in which they were embedded” (333).

## Methods

This paper examines sense of belonging for immigrant and Canadian-born populations in small-to-medium sized Canadian urban areas. Following Research Ethics approval from McMaster University, data was collected from three sites: a) Charlottetown, Prince Edward Island; b) Hamilton, Ontario, and c) Saskatoon, Saskatchewan. These three sites were chosen as they represent three of Canada’s major geographical regions (Atlantic, Central, and Western) and provide the opportunity to compare immigrant experiences in cities with varying levels of economic renewal and different sized immigrant populations. Following Tashakkori & Teddlie (Tashakkori & Teddlie 2003), a sequential mixed methods approach was employed, involving a (1) telephone survey and (2) focus group sessions in each city.

A comprehensive household survey (comprising 73 questions) was administered by a Saskatoon-based research consulting firm via telephone to a random selection of households in Charlottetown, Hamilton and Saskatoon (Census Subdivision) between May and August, 2012. These survey questions have been tested and validated over the past 10 years in several Canadian city contexts to ensure that they are an accurate reflection of residents’ perceptions of QOL (Kitchen et al. 2012b; Kitchen & Williams 2010; Muhajarine et al. 2008; Williams et al. 2008; Randall et al. 2008). The survey asked respondents questions related to a number of topics including perceptions of QOL, neighbourhood and city conditions, health and belonging, sense of place, and respondents’ socio‐demographic information. The variable named ‘sense of belonging to local community’ was used as a proxy for ‘sense of belonging’, given that we wanted to be sure that the immigrant respondents were cognizant of what was meant by belonging, irrespective of which community they affiliated themselves with (e.g. ethnic, host population). The sample consisted of randomly selected households where the primary respondent was at least 18 years old and was either Canadian-born or an immigrant (defined as a person who was not born in Canada). A total of 1529 surveys were completed, including 413 (27 %) by immigrant respondents. The overall response rate was 23 %. Prior to data collection, power calculations were performed to determine the appropriate sample sizes in the three cities including the proportions of immigrants. The 2006 Census was employed to aid in these calculations by determining the total number of households and estimating the number of immigrant households in each of the three cities. The proportion of immigrants in the telephone sample (27 %) is in line with the levels found for the three study cities in the 2011 National Household Survey (NHS) conducted by Statistics Canada. Level of education, marital status, employment status, housing tenure and, for immigrant respondents, years lived in Canada, were found to be reflective of data from the 2011 NHS.

The telephone survey data informed the collection of the qualitative data. Late in 2012 and early in 2013, a total of 11 audiotaped focus groups were conducted by the research team, with the help of translators in some cases, in the three cities. The focus group participants were not a subset of the telephone survey sample, but rather were purposively recruited, based on ethnicity and residential longevity (details below), from a range of immigrant community organizations within each city. Community partner organizations in each city recruited participants, who were reimbursed $25CND for their participation. Table 2 provides details of the focus groups; approximately 7 participants were in each focus group. Given the large proportion of Mandarin-speaking Chinese immigrants in each of the three city sites, this group participated in two focus group discussions in each city (*n* = 6). The first was conducted in Mandarin with immigrants who have resided in Canada between 1 and 5 years. The second was conducted in English with immigrants who have resided in Canada between 6 and 10 years. An additional immigrant group was selected in each city for a focus group. In Charlottetown, the additional focus group was conducted with Farsi-speaking Iranians while in Saskatoon it was held with Tamil-speaking South Asians. In Hamilton an additional focus group was comprised of Urdu-speaking Pakistanis and due to cultural norms, separate male and female sessions were held. All the non-English focus groups were translated into English before being thematically analysed. The themes were generated using line-by-line coding and all qualitative analysis was (*n* = 3) performed by the same researcher. Community stakeholder focus groups were also held in each city to crosscheck the preliminary results. Participants include representation from a wide range of municipal government and NGO organizations, who largely confirmed the results of the research, providing directions for policy and program implications. In this paper, the focus groups were used to augment the telephone survey data. The focus group discussions with immigrants demonstrated a positive sense of belonging to community, although there was some indication of hidden bias when issues of employment and social inclusion were discussed.

**Table 2:** Focus group socio-demographic characteristics

Location	Charlottetown	Saskatoon	Hamilton & Saskatoon	All sites	All sites
Country/region of origin	Iran (*n* = 1)	South Asia (*n* = 1)	Pakistan (*n* = 3)	Chinese (*n* = 3)	Chinese (*n* = 3)
Language of focus group	Farsi	Tamil	Urdu	Mandarin	English
Number of participants	7	5	18	19	18
Gender					
Male	4	1	9	11	8
Female	3	4	9	8	10
Employment Status					
Employed	1	5	4	7	14
Unemployed	6	0	11	6	0
Self-Employed	0	0	0	1	2
Retired	0	0	1	3	1
Other	0	0	2	2	1
Average years lived in city/province	2.3	4	2.9	1.8	11.8

The telephone survey data was analyzed using the statistical software Stata 13 and involved two steps. The first was the use of descriptive statistics by way of bar charts and contingency tables to measure the relationship between sense of belonging and a number of independent variables. The ‘lincom’ command (linear combinations of estimators) in Stata was used to compare the proportions displayed in Figs. 3 and 4 and to test for statistical significance. The second step involved the use of ordered logistic regression modeling. As described by Kitchen et al (Kitchen et al. 2012a) compared to ordinary least squares (OLS), this technique is more appropriate given the categorical nature (4 point Likert scale) of the dependent variable: sense of belonging to local community. The ordered logistic regression model more appropriately accounts for nuanced differences across the categorical scale variable and controls for the constraints of the data; neither logistic or OLS address these issues sufficiently. Further, the ordered logistic model allows for a more parsimonious presentation of output given the proportional odds assumption (e.g. parallel regressions), compared to more generalized models with few restrictions. Odds ratios compare the probability of events for two groups, where an odds ratio of 1 implies an event that is equally likely to occur in one group as it is in the other group. An odds ratio greater than 1 implies the event is more likely to occur in the comparison group than the reference group. Further, an odds ratio less than 1 means the event is less likely in the comparison group than the reference group. The selection of a reference group is required in logistic regression and is normally the category within the independent variable that has the highest count. For example, the independent variable housing tenure (see Table 3) consists of two categories: 1) renting and 2) owning with owning having the highest count and therefore designated the reference category. All of the statistical analysis in the paper was conducted using tests of statistical significance (95 % confidence level).

**Table 3:** Independent variables: Health and Socio-Demographic Conditions 2012 Quality of Life Telephone Survey

Variable	Survey question	Coded responses
Self-perceived health	In general, would you say your health is?	1. Excellent
		2. Very good
		3. Good
		4. Fair/poor
Self-perceived mental health	In general, would you say your mental health is?	1. Excellent
		2. Very good
		3. Good
		4. Fair/poor
Household income	What is your total annual income before taxes?	1. Less than $20,000
		2. $20,000 to $39,999
		3. $40,000 to $79,999
		4. $80,000 and more
		5. Not stated
Housing tenure	Do you or a member of your household own or rent the dwelling you live in?	1. Own
		2. Rent
Marital status	What is your marital status?	1. Single/never married
		2. Married/common law
		3. Separated/widowed/divorced
Education	What is your current level of education?	1. Less than high school
		2. High school
		3. Some post-secondary
		4. College or trades
		5. University
Employment status	During the past 12 months were you mainly…?	1. Working full-time
		2. Working part-time
		3. Unemployed
		4. Retired
		5. Other
Living arrangement	Which of the following best describes your current living arrangement?	1. Unattached alone
		2. Unattached with others
		3. Couple with children
		4. Couple alone
		5. Single parent/other
Age	What is your age group?	1. Age 18 to 24
		2. Age 25 to 44
		3. Age 45 to 64
		4. Age 65 and over
City	Place where respondent resides	1. Charlottetown
		2. Hamilton
		3. Saskatoon
Years lived in Canada	Immigrants: How many years have you lived in Canada?	1. 5 years or less
		2. 6 to 10 years
		3. More than 10 years

The focus group information was first analyzed using manual coding; thematic coding was used to identify factors that enhance and detract from sense of belonging. A text search query for the words ‘belong’ and ‘discriminate’ (including stemmed words) was conducted using NVIVO 9. In the next section, the results of the qualitative stage (focus groups) are integrated into the main findings of the quantitative research (telephone survey) in order to better express the notions of belonging emanating from the two data sets.

It is important to point out a major difference in the two methods of analysis. The focus groups were comprised entirely of visible minority immigrants representing different ethnic groups in the three study sites. The telephone survey does not distinguish between ethnic groups but rather includes immigrants in one category. There are three reasons for this. The first is that while the telephone survey asked a question about visible minority status (yes or no), an extensive analysis of the data showed no significant differences (in terms of sense of belonging and other indicators) between immigrants who are visible minorities and those who are not. Second, the large majority of immigrants to Canada over the past 10 years (more than 80 %) have been members of a visible minority group and this is also reflected in the responses from the telephone survey. Third, due to cost constraints it was not possible to include an ‘open-ended’ answer to a survey question asking respondents to identify their ethnic origin. Ethical approval for this study was obtained from the McMaster University Research Board. Written informed consent was obtained for participation in this study.

## Results

### Summary statistics

**Fig. 1:**
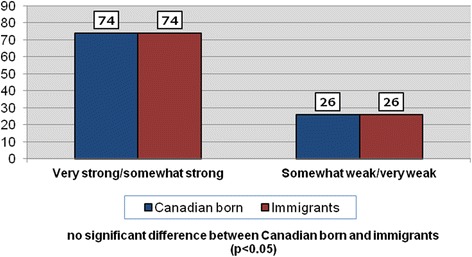
Sense of belonging to local community among respondents (%) (all sample: *n* = 1529)

**Fig. 2:**
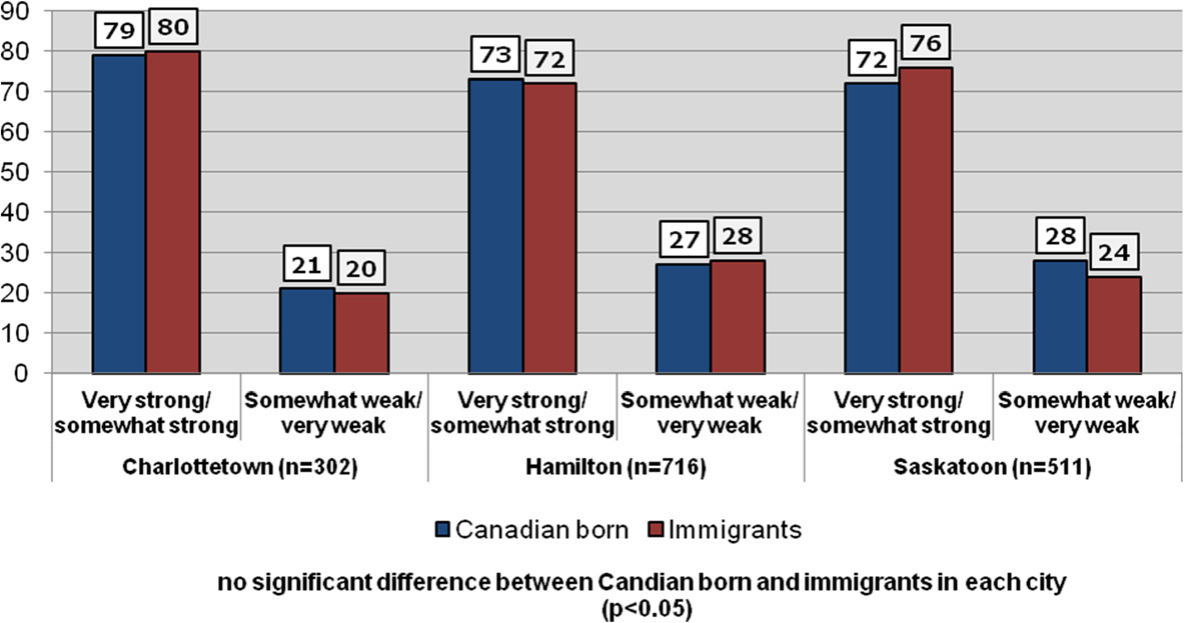
Sense of belonging to local community among respondents (%)

Table 4 shows the distribution of the responses of the dependent variable. When asked, ‘how would you describe your sense of belonging to your local community?’, just 7 % of respondents indicated that it is ‘very weak’, while 56 % replied that it is ‘somewhat strong’ and a further 16 % said that their sense of belonging is ‘very strong’. Figure 1 compares two categories of responses to this question across Canadian-born and immigrant respondents. The chart shows that identical proportions of Canadian-born and immigrants rated their sense of community belonging (SoCB) as either ‘very strong/somewhat strong’ (74 %) or ‘somewhat weak/very weak’ (26 %). It is encouraging that the large majority of respondents have a positive SoCB. Figure 2 displays levels of SoCB in the three study cities. Charlottetown has the highest rates SoCB (very strong/somewhat strong) and, similar to the overall sample, are virtually identical between Canadian-born (79 %) and immigrants (80 %). While positive perceptions of SoCB are slightly lower in Hamilton and Saskatoon, there are again no significant differences in levels between the two groups.

**Table 4:** The dependent variable: Sense of Belonging 2012 Quality of Life Telephone Survey

How would you describe your sense of belonging to your local community?			
Response	Code	Frequency	Percent
Very weak	1	105	7.1
Somewhat weak	2	283	19.0
Somewhat strong	3	857	57.7
Very strong	4	241	16.2
Total		1,486	100

These findings were reflected in the focus groups with immigrants where participants expressed an overall feeling of belonging. A Hamilton participant acknowledged feelings of satisfaction with the environment and the community:

*“I think I feel the sense of belonging. The environment is good. There are many activities that I can join and the community takes care of us. I’m satisfied with where I live.”‐*Hamilton Participant (Recent immigrant, Mandarin-speaking Chinese)

A Saskatoon participant expressed feelings of belonging and comfort:

*“Definitely, going to other cities, it is nice to look around…but you don’t feel that that is home…the thought of, oh I am living in Saskatoon, is a happy feeling…when you go to big cities especially, it is very nice…you can stay there for 2 days…if you ask yourself if you are able to live there for a whole lifetime…you think, no, I like going back to Saskatoon…I belong there…like that…” -*Saskatoon Participant (Recent immigrant, Tamil-speaking South Asian)

Similarly, a Charlottetown participant described her feeling of ‘being home’:

*“Like being in China, in PEI there is a little sense of belonging, each time you leave the province, just after a few days; I really want to go back to PEI. Once I cross the bridge, I have the feeling of being home”. –*Charlottetown Participant (Recent immigrant, Mandarin-speaking Chinese)

Participants also acknowledged the feelings of belonging associated with smaller urban centres:

*“With everyone, there’s that warmth, that friendliness…in a bigger city, that is a challenge. They mind their own business. It’s not like that here. Everyone is welcoming. They give you that sense of belonging. And that is really important, regardless of the city”. -* Saskatoon Participant (Recent immigrant, Tamil-speaking South Asian)

In addition to expressing feelings of belonging to their local community, participants expressed a strong sense of belonging within their ethnic community. For example, a Charlottetown participant highlighted the importance of relationships within an ethnic community:

*“For me, family is one. And also the Chinese community is another one. I think I found I have a strong sense of belonging. We get together and I feel that, if I have a need, I can go to, kind of go to them, count on them; and also few, very few local friends that I was able to fortunately form relationship … Same, most of them are from work…” -*Charlottetown Participant (Established immigrant, English-speaking Chinese)

Similarly, a Saskatoon participant acknowledged the strong attachment to one’s ethnic background:

*“I do not have a strong sense of belonging at this stage. This perhaps has to do with my travel experience. My wife and I travel to different places every year. So our real sense of belonging is in Taiwan because it is where our home and ancestry are”. -*Saskatoon Participant (Recent immigrant, Mandarin-speaking Chinese)

**Fig. 3:**
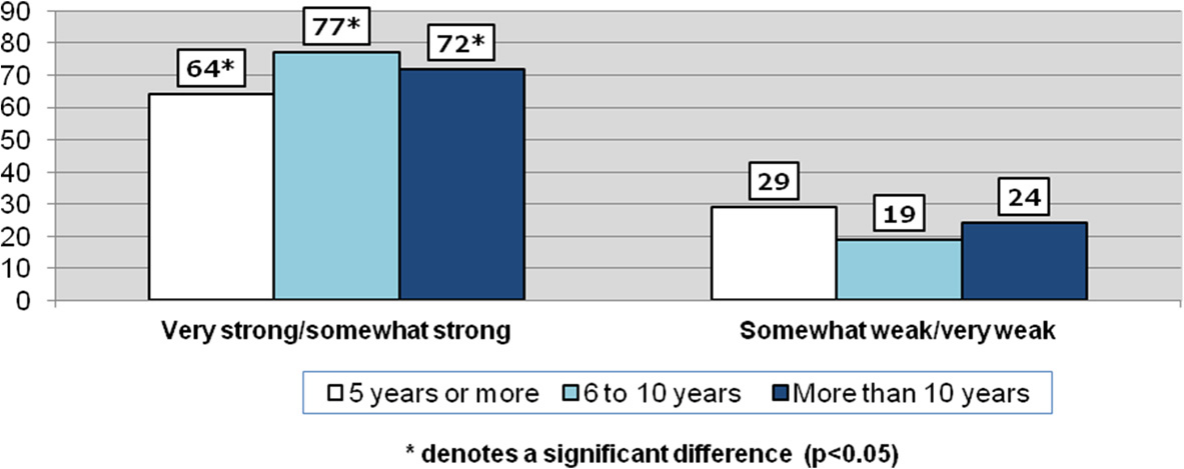
Sense of belonging to local community among immigrants by years lived in Canada (%) (*n* = 413)

**Fig. 4:**
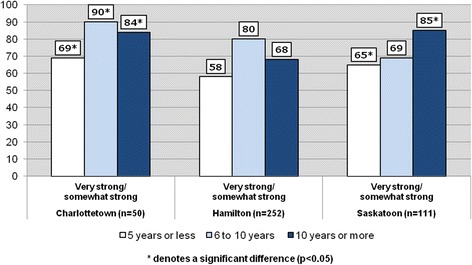
Sense of belonging to local community among immigrants by years lived in Canada (%)

In Figs. 3 and 4, the telephone survey sample is restricted to immigrant respondents (*n* = 413) and SoCB is measured according to the length of time lived in Canada. In keeping with the literature, Fig. 3 reveals that positive perceptions increase over time. Among newcomers who have lived in the country for 5 years or less, 64 % said that they have a ‘very strong/somewhat strong’ SoCB with this proportion rising to 77 % among those who have resided in Canada from 6 to 10 years before dropping slightly to 72 % for immigrants living in the country 10 years or more. These proportions were statistically significant across the three time-periods. A similar trend is visible among immigrants in the three cities, with positive SoCB increasing over time. Figure 4 indicates that immigrants in Charlottetown who have lived in Canada between 6 and 10 years have a high positive SoCB (90 %). However, while positive SoCB increases over time for immigrants in Hamilton, the proportions were found to be not statistically significant between the three time periods. A significant increase in SoCB is found in Saskatoon between immigrants who have lived in Canada for 5 years or less (65 % reporting a positive SoCB) and those who have resided in the country for 10 years or more (85 % indicating a positive SoCB).

The following observation from a Hamilton focus group participant indicates an increase in feelings of belonging over time:

*“I still don't have much feeling of belonging, but it is a lot better than when I was first arrived. I was feeling really insecure. I don’t know why. Probably because my parents are not here with me… and my husband only came a few times. I hoped I had a job and I wouldn’t be feeling like floating on water. I feel much better now, since I started to go to school and made some friends. Now I have a little feeling of belonging”. –*Hamilton Participant (Recent immigrant, Mandarin-speaking Chinese)

### Ordered regression analyses: health and socio-demographic conditions

A series of regression models were produced to examine the association between SoCB and 1) health and socio-demographic conditions and 2) community perceptions. The dependent variable, SoCB and its coded responses, is shown in Table 4. Table 3 lists the 11 health and socio-demographic variables, which serve as the independent variables. They include self-perceived health and mental health, income, housing, marital status, education, employment, living arrangement, age, city of residence and, for immigrant respondents, years lived in Canada. These independent variables are indicative of the factors which may influence belonging. The coding of each is displayed in Table 3. For several variables, including self-perceived health and mental health, the ‘fair’ and ‘poor’ categories had to be combined into a single category ‘fair/poor’ to ensure a sufficient count.

Table 5 displays the results of the ordered regression according to 3 models: 1) all respondents (*n* = 1486); 2) Canadian-born (*n* = 1092), and; 3) immigrants (*n* = 394). The objective is to determine what socio-demographic differences exist among the three groups in influencing a positive increase in SoCB – according to responses across the 4-point scale (it is important to note that ordered logit regression measures positive change across the SoCB scale with code 1 signifying the lowest belonging and code 4 indicating the highest). In Model 1, several variables are shown to have a significant association with SoCB. Respondents indicating that they have ‘excellent’ mental health are more likely (OR = 1.427, CI: 1.09–1.85) to have a positive increase in their SoCB compared to those with ‘very good’ mental health (the reference group). At the same time, respondents saying they have ‘good’ (OR = 0.651, CI: 0.49–0.86) or ‘fair/poor’ (OR = 0.505, CI: 031–0.83) mental health are less likely to have a positive increase in their SoCB. Interestingly, among all respondents, those working part-time are more likely (OR = 1.559, CI: 1.03–2.34) to experience an improvement in their SoCB compared to the reference group, those in the ‘other’ category.

**Table 5:** Results of Ordinal logit regression analyses of sense of belonging to local community^a^ (Health and socio-demographic conditions)

	Model 1		Model 2		Model 3	
	All respondents		Canadian-born		Immigrants	
Independent variables	Odds ratios	95 % CI	Odds ratios	95 % CI	Odds ratios	95 % CI
Self-perceived health						
Excellent	1.261	0.93–1.70	1.15	0.81–1.64	1.63	0.90–2.98
Very good	Reference		Reference		Reference	
Good	0.753^c^	0.56–0.95	0.696^c^	0.50–0.96	0.717	0.43–1.20
Fair/poor	0.823	0.56–1.19	0.700	0.45–1.07	1.204	0.54–2.69
Self-perceived mental health						
Excellent	1.427^d^	1.09–1.85	1.413^c^	1.03–1.92	1.488^c^	0.87–2.55
Very good	Reference		Reference		Reference	
Good	0.651^d^	0.49–0.86	0.671^c^	0.48–0.93	0.581^c^	0.33–1.01
Fair/poor	0.505^d^	0.31–0.83	0.634	0.34–1.15	0.228^d^	0.09–0.59
Household Income						
Less than $20,000	0.853	0.57–1.27	0.825	0.50–1.34	1.055	0.49–2.23
$20,000 to $39,999	0.669^c^	0.47–0.94	0.670^a^	0.44–1.01	0.638	0.33–1.20
$40,000 to $79,999	Reference		Reference		Reference	
$80,000 or more	1.101	0.82–1.47	1.116	0.79–1.56	1.498	0.77–2.89
Not stated	0.961	0.70–1.30	0.950	0.66–1.36	1.062	0.57–1.98
Housing tenure						
Own	Reference		Reference		Reference	
Rent	0.884	0.68–1.15	0.996	0.72–1.37	0.564^d^	0.32–0.98
Marital status						
Single/never married	0.697	0.34–1.40	0.897	0.36–2.18	0.442	0.12–1.61
Married/common law	Reference		Reference		Reference	
Separated/widowed/divorced	0.394^c^	0.19–0.80	0.458	0.18–1.11	0.355	0.09–1.35
Education						
Less than high school	1.521	0.94–2.45	1.32	0.75–2.33	2.262	0.87–5.88
High school	0.866	0.64–1.17	0.847	0.58–1.22	0.947	0.51–1.73
Some post-secondary	0.917	0.66–1.26	0.872	0.59–1.28	0.948	0.50–1.79
College or trades	0.887	0.67–1.16	0.876	0.64–1.19	0.984	0.54–1.76
University	Reference		Reference		Reference	
Employment status						
Working full-time	0.972	0.69–1.35	0.724	0.48–1.08	2.066^d^	1.10–3.85
Working part-time	1.559^c^	1.03–2.34	1.370	0.82–2.27	1.988^c^	0.97–4.06
Unemployed	1.227	0.66–2.26	1.187	0.54–2.58	1.640	0.56–4.72
Retired	1.404	0.89–2.19	1.09	0.64–1.86	2.730^c^	1.11–6.67
Other	Reference		Reference		Reference	
Living arrangement						
Unattached alone	2.022	0.97–4.19	1.544	0.63–3.84	2.746	0.68–10.92
Unattached with others	1.323	0.65–2.71	1.066	0.43–2.62	1.616	0.43–5.96
Couple with children	0.978	0.73–1.30	0.921	0.65–1.29	0.827	0.45–1.51
Couple alone	Reference		Reference		Reference	
Single parent/other	1.733	0.79–3.76	1.501	0.57–3.97	1.575	0.37–6.63
Age						
18-24 years	0.828	0.52–1.31	0.690	0.39–1.20	1.211	0.47–3.11
25-44 years	0.687^d^	0.52–0.89	0.729^c^	0.53–0.99	0.622	0.34–1.12
45-64 years	Reference		Reference		Reference	
65 years and over	1.299	0.89–1.88	1.644^c^	1.06–2.53	0.594	0.27–1.26
City						
Hamilton	Reference		Reference		Reference	
Charlottetown	1.243	0.94–1.63	1.219	0.89–1.66	1.628	0.84–3.15
Saskatoon	0.873	0.69–1.10	0.797	0.60–1.05	1.387	0.80–2.39
Years lived in Canada						
5 or less	Reference		Reference		Reference	
6 to 10	--		--		1.998	0.82–4.83
10 or more	--		--		1.249	0.60–2.58
Observations	1,486		1,092		394	
Pseudo R2	0.0469		0.0474		0.0890	

^a^The dependent variable is sense of belonging to local community using a 4 point scale: 1-very weak, 2-somewhat weak, 3-somewhat strong, 4-very strong. The model used for estimation is Ordered Logit. Confidence intervals indicated. (^b^) significant at 10 %, (^c^) significant at 5 %, (^d^) significant at 1 %. Reference categories are included in the table

In Model 2 (Table 5), the sample is restricted to Canadian-born respondents and two independent variables are noteworthy in their association with SoCB. First, respondents with ‘excellent’ mental health are more likely (OR = 1.413, CI: 1.03–1.92) to experience a positive increase in their SoCB then those with ‘very good’ mental health (the reference group). Second, Canadian-born seniors are more likely (OR = 1.644, CI: 1.06–2.53) to have an improving SoCB than people aged 45 to 64 (the reference group).

Model 3 includes the immigrant population only and a different set of associations emerges. Like the overall and Canadian-born samples, ‘excellent’ mental health among immigrants (OR = 1.488, CI: 0.87–2.55) is associated with improving SoCB. However, two additional socio-demographic conditions are related to SoCB. The first is housing tenure, with immigrants who rent being significantly less likely (OR = 0.564, CI: 0.32–0.98) to have a positive increase in SoCB compared to immigrants who own a home (the reference group). The second is employment status, with immigrants who are working full-time more likely (OR = 2.066, CI: 1.10–3.85) to have an increasing SoCB compared to immigrants in the ‘other’ (disabled/student/homemaker) category (the reference group). Furthermore, immigrants who are working part-time (OR = 1.988, CI: 0.97–4.06) and who are retired (OR = 2.730, CI: 1.11–6.67) are also more likely to have an increasing SoCB.

The following focus group observation is indicative of the relationship between owning a home and feelings of belonging:

*“I don’t think I have the feeling of belonging, because I don’t own a house or a car (no home).”-*Hamilton Participant (Recent immigrant, Mandarin-speaking Chinese)

In addition, the issue of employment was regularly discussed in the focus groups; in Charlottetown, discrimination at work is reflected in the following observation:

*“I work at the xxxx, especially on this topic- when a customer is in line, they should have their turn for services, but he [the customer] would rather continue to wait, so that a counter person at the xxxx who is local, is able to serve him. Some people’s speaking attitude is not very friendly. If there is no problem with the service, then it is fine, but if there is a little bit of a problem, they immediately will give you a bad face, very ugly, and be impolite. It has little effect on my quality of life. But if they do not change their attitude, we can’t integrate.” ‐*Charlottetown Participant (Recent immigrant, Mandarin-speaking Chinese)

A Hamilton participant showed frustrations with employment limitations:

*“Here you try to melt into this society, but you always feel some limitations. Maybe, it’s not kind of discrimination, but anything you don’t feel good. I got my Ph.D. in (university’s name is not clear) in U.S.A. I think I got some experiences working in United States for several years. I know something about North American, cultural or something like that, but I still feel that I’m not wholly, totally accepted by this society. You know it’s hard. It’s kind of a sense. You don’t have a sense of belonging, but you can’t figure out. You can’t pick up, oh, this is an example, that is an example, you can’t see clearly, but you really got this feeling. This feeling is potential.” –*Hamilton Participant (Established immigrant, English-speaking Chinese)

### Ordered regression analyses: community perceptions

The quality of life survey also included several questions where respondents were asked their views on neighbourhood and community issues. Five of these questions were employed as independent variables to assess their influence on SoCB. The variables are listed in Table 6 and include perceptions of schools, recreation programs, neighbours, socializing and trust, all community related factors likely to be associated with belonging. The coding of each is shown in Table 6. Table 7 shows the results of the regression analysis with 3 models again being constructed. In Model 1 (the entire sample), several community perceptions are found to have an association with improving SoCB. Respondents who rate the quality of schools in their neighbourhood as ‘excellent’ are more likely (OR = 1.681, CI: 1.17–2.39) to have a positive increase in SoCB compared to people who rated the quality of schools in their neighbourhood as ‘good’ (the reference group). Similarly, respondents who rate the quality of recreation programs and services in their neighbourhood as ‘excellent’ are more likely (OR = 1.759, CI: 1.18–2.60) to have a positive increase in SoCB compared to those who rated these programs and servives as ‘good’ (the reference group). For all respondents, knowing their neighbours (OR = 2.336, CI: 1.62–3.36), and participating with them in social activities (OR = 1.841, CI: 1.36 = 2.49) are also significantly associated with an improving SoCB. When the sample includes Canadian-born respondents only (Model 2), an almost identical set of predictors emerges. Again, the quality of schools (OR = 1.604, CI: 1.06–2.42) and recreation programs (OR = 2.394, CI: 1.53–3.72), knowing their neighbours (OR = 2.260, CI: 1.45–3.50) and participating in social activities with them (OR = 1.987, CI: 1.3–2.82), are all significantly associated with SoCB.

**Table 6:** Independent variables: Community Perceptions 2012 Quality of Life Telephone Survey

Variable	Survey question	Coded responses
Schools	How do you rate the quality of schools in your neighbourhood?	1. Excellent
		2. Very good
		3. Good
		4. Fair/poor
		5. Not sure
Recreation	How do you rate the quality of recreation programs and services in your neighbourhood?	1. Excellent
		2. Very good
		3. Good
		4. Fair/poor
		5. Not sure
Neighbours	How true is the following statement? I know many of my neighbours on a first name basis.	1. Very true
		2. Fairly true
		3. Neutral
		4. Not very true
		5. Not at all true
Socialize	How often do you participate in social activities with your neighbours?	1. All the time/often
		2. Sometimes
		3. Hardly ever
		4. Never
Trust	Do you agree that most people can be trusted?	1. Very much
		2. Somewhat
		3. A little
		4. Not much

**Table 7:** Results of ordinal logit regression analyses of sense of belonging to local community^a^ (Community Perceptions)

	Model 1		Model 2		Model 3	
	All respondents		Canadian-born		Immigrants	
Independent variables	Odds ratios	95 % CI	Odds ratios	95 % CI	Odds ratios	95 % CI
Quality of schools in neighbourhood						
Excellent	1.681^d^	1.17–2.39	1.604^c^	1.06–2.42	2.156^c^	1.04-4.45
Very good	1.212	0.91–1.61	1.191	0.84–1.67	1.555	0.87–2.75
Good	Reference		Reference		Reference	
Fair/poor	0.700	0.48–1.02	0.754	0.48–1.18	0.545	0.26–1.13
Not sure	0.831	0.61–1.11	0.830	0.58–1.18	1.00	0.57–1.76
Quality of recreation programs in neighbourhood						
Excellent	1.759^d^	1.18–2.60	2.394^d^	1.53–3.72	0.676	0.26–1.73
Very good	1.051	0.80–1.36	1.231	0.90–1.67	0.750	0.42–1.32
Good	Reference		Reference		Reference	
Fair/poor	0.596^d^	0.43–0.81	0.542^d^	0.36–0.80	0.651	0.37–1.13
Not sure	0.783	0.54–1.13	0.856	0.54–1.35	0.617	0.32-1.18
Know neighbours on first name basis?						
Very true	2.336^d^	1.62–3.36	2.260^d^	1.45–3.50	2.976^d^	1.48–5.97
Fairly true	1.433^c^	1.01–2.01	1.406	0.93–2.12	1.875^c^	1.00–3.51
Neutral	Reference		Reference		Reference	
Not very true	1.144	0.75–1.72	1.316	0.79–2.19	0.917	0.44–1.88
Not at all true	0.920	0.58–1.45	1.024	0.58–1.80	0.982	0.43–2.21
Participate in social activities with neighbours?						
All the time/often	1.841^d^	1.36–2.49	1.987^d^	1.39–2.82	1.299	0.70–2.40
Sometimes	Reference		Reference		Reference	
Hardly ever	0.778^b^	0.58–1.03	0.714^c^	0.51–0.99	0.969	0.54–1.73
Never	0.345^d^	0.25–0.46	0.275^d^	0.18–0.40	0.457^d^	0.26–0.79
Agree that most people can be trusted?						
Very much	1.216	0.95–1.55	1.006	0.75–1.33	1.819^c^	1.08–3.05
Somewhat	Reference		Reference		Reference	
A little	0.750^b^	0.55–1.01	0.720^b^	0.50–1.03	0.829	0.46–1.47
Not much	0.648^c^	0.44–0.94	0.516^d^	0.33–0.80	1.145	0.56–2.32
City						
Hamilton	Reference		Reference		Reference	
Charlottetown	0.953	0.71–1.26	0.994	0.71–1.37	0.962	0.49-1.87
Saskatoon	0.894	0.70–1.12	0.814	0.61–1.07	1.758^c^	1.04-2.94
Years lived in Canada						
5 or less	Reference		Reference		Reference	
6 to 10	--		--		1.450	0.62–3.37
10 or more	--		--		1.654^c^	0.93–2.91
Observations	1,486		1,092		394	
Pseudo R2	0.1077		0.1263		0.1005	

^a^The dependent variable is sense of belonging to local community using a 4 point scale: 1-very weak, 2-somewhat weak, 3-somewhat strong, 4-very strong. The model used for estimation is Ordered Logit. Confidence intervals indicated. (^b^) significant at 10 %, (^c^) significant at 5 %, (^d^) significant at 1 %. Reference categories are included in the table

Model 3 is restricted to immigrant respondents. It is interesting to note that for this group, the quality of schools in the neighbourhood appears to be more strongly associated with SoCB then the entire sample or the Canadian-born. Immigrants who feel that the quality of schools is ‘excellent’ are more likely (OR = 2.156, CI: 1.04–4.45) to have an increasing SoCB compared to the reference group (those who said the quality of schools are ‘good’). This is reflected in the experience of one of the focus group participants, who said:

*“My child goes to school here… so I force myself to have a sense of belonging in Saskatoon. I tell myself I belong here. I force myself to think this way. I feel time will change everything. I will not have to force myself as time goes by. [Laughter].” –*Saskatoon Participant (Recent immigrant, Mandarin-speaking Chinese)

According to the regression analysis, the quality of recreation programs does not appear to be associated with SoCB. Nevertheless, the importance of services and recreation programs was highlighted in the focus group discussions:

*“We have all kinds of religious freedom and there is a mosque where we pray. There are also clubs for seniors and I have friendship in the clubs and organizations in Hamilton. These are 2–3 main factors. In Hamilton we do have a sense of belonging”. –Hamilton Participant* (Recent male immigrant, Urdu-speaking Pakistani)

However, as shown in Table 7, knowing their neighbours on a first name basis is more significantly associated with an improving SoCB among immigrants than it is for the Canadian-born population (OR = 2.976, CI: 1.48–5.97). It is evident that trusting people in general is also more significant for immigrants (OR = 1.819, CI: 1.08–3.05). In addition to these community perceptions, an increase in SoCB is also associated with immigrants who live in Saskatoon (OR = 1.758, CI: 1.04–2.94) and those who have lived in Canada for 10 years or more (OR = 1.654, CI: 0.93–2.91).

## Discussion

By comparing the Canadian-born population to immigrants living in three small-to-medium sized Canadian urban areas, we have learned that there is more similarity than difference between these two groups. The results provide useful insights into the quality of life of a segment of the Canadian population (namely immigrants in smaller urban areas) that has often been overlooked in a body of research focused primarily on the country’s largest urban areas. With respect to the role of time in SoCB evaluations, the data reflect what is known in the literature, specific to immigrant respondents having increased positive perceptions of SoCB over time.

This paper has made a unique contribution from earlier work conducted by Kitchen et al. (Kitchen et al. 2012a), which examined broad regional trends in sense of belonging in Canada by way of a large national survey, the CCHS. By comparison, the current paper analyzed in more detail sense of community belonging in three small-to-medium sized urban areas by way of a validated telephone survey and a mixed methods approach incorporating focus groups with immigrants. Several of the dimensions captured in the current paper, specifically community perceptions (e.g. quality of schools and recreation, knowing your neighbours and trusting people) were not dealt with in the earlier work nor were the unique observations from immigrants themselves (by way of the focus groups).

The paper found that SoCB was very high among all respondents. About three-quarters (74 %) rated their belonging as ‘very strong/somewhat strong’. Encouragingly, levels of belonging were almost identical between immigrants and Canadian-born respondents not only in the entire sample but also in the three study sites – Charlottetown, Hamilton and Saskatoon. No statistically significant differences were found between immigrants and Canadian-born residents in these cities. It is possible that the high level of belonging observed among the immigrant population is a combination of two distinct forms of belonging, reflected in their dual identities: belonging to one’s ethnic community, and belonging to wider Canadian society. Building on the instructive work of Antosich (Antonsich 2010), Gilmartin (Gilmartin 2008) and Huot (Huot et al. 2014) on the scales and politics of belonging, further research is needed to investigate the intersection of place, geography and migration on overall feelings of community belonging, as well as the impact this has on immigrants’ physical and mental wellbeing.

The qualitative analysis (focus groups) demonstrated that, in general, immigrants have a strong sense of belonging to both their city of residence and to their own ethnic community. As shown in Table 8, there are a number of subtle differences within this overall trend. For example, both the Iranian and Tamil speakers expressed feelings of belonging to the wider Canadian society. Hamilton’s Urdu-speaking group also acknowledged a strong sense of belonging; however, this was attributed to Hamilton’s large Pakistani population.

**Table 8:** Thematic comparison across focus groups

City	Group	LOR	General Feelings of Belonging
Charlottetown	Chinese	Established	• Strong islander mentality hinders immigrant sense of belonging
			• Strong sense of belonging within the Chinese community
			• Employment contributes to sense of belonging; respondents feel that they belong among co-workers
		Recent	• Also mentioned the strong islander mentality; feelings that immigrants are not welcome
			• Identify with Chinese community
			• Some mention of PEI as “home”
	Iranian	Recent	• Immigrants feel like they belong in Canadian society
			• Belonging is attributed to feelings of security, peace, and the hospitable nature of residents
Hamilton	Chinese	Established	• Feelings of discrimination
			• Language acknowledged as a barrier to belonging
			• Length of residence contributes to belonging; increased attachment to Hamilton over time
		Recent	• Strong ethnic identity
			• Weak sense of belonging
			• Sense of belonging increasing over time; respondents are optimistic about their future in Hamilton
	Urdu (female)	Recent	• Feel excluded from community events; celebrate religious events within ethnic community
			• Strong sense of belonging within Hamilton due to large Pakistani population
	Urdu (male)	Recent	• Appreciative of religious freedom
			• Again, sense of belonging within Pakistani community
			• Increase in sense of belonging over time
Saskatoon	Chinese	Established	• Feeling that Saskatoon is “home”
		Recent	• House and family are important contributors to feelings of belonging
			• Sense of belonging seems to be forced
	Tamil	Recent	• Appreciative of smaller-sized city; familiarity contributes to belonging
			• Feelings of belonging to wider society, not just within ethnic community
			• Employment contributes to belonging
	Urdu	Recent	• Appreciative of religious freedom
			• General feeling that Canadian-born individuals are “nice”
			• Feelings of discrimination regarding recognition of credentials

However, the paper did find that SoCB improves among immigrants who have lived in Canada for longer periods of time. Immigrants residing in Canada for five years or less had significantly lower levels of SoCB compared to those who have lived in the country for 6 years or more. This trend was evident among longer-term immigrants (more than 10 years) in Charlottetown and particularly in Saskatoon who reported very high levels of SoCB (about 85 % having a ‘very strong/somewhat strong’ SoCB).

Focus group participants also expressed increased feelings of belonging to their local community over time, as depicted by the quote on page 15 from the Hamilton Mandarin speaking Chinese participant. Though not explicitly mentioned in all focus group discussions, the increase in sense of belonging over time is apparent through the comparison of recent and established Chinese immigrants across the three sites (Table 8). For example, recent immigrants display a stronger attachment to their Chinese community, which may hinder belonging to the wider host society. As shown on page 17, a recent Chinese immigrant to Saskatoon feels a forced sense of belonging. In comparison, established Chinese immigrants described Saskatoon as their home. As suggested in research by Reitz & Banerjee (Reitz & Banerjee 2007), Huot et al (Huot et al. 2014) and others, discrimination and feelings of exclusion may influence an immigrant’s stronger sense of attachment to their own community and relates to the question posed by Antonisch (Antonsich 2010) on the creation of communities of belonging beyond communities of identity.

In terms of health and socio-demographic factors and consistent with the literature (Choenarom et al. 2005; Hagerty & Williams 1999) the regression analysis found that positive mental health was associated with improving SoCB in the entire survey sample, as well as in the Canadian-born and immigrant samples. It is interesting to note that in addition to mental health, improving SoCB among immigrants was strongly associated with full-time employment and home ownership, as reflected in the qualitative data. By comparison, neither of these factors had an influence among Canadian-born respondents.

As is evident in many of the quotes presented abovce, participants in all three cities expressed tensions in belonging to their new cities – even though not specifically asked via the focus group schedule. In line with the findings of Fontana (Fontana 2003), Sonn (Sonn 2002) and Reitz and Banerjee (Reitz & Banerjee 2007), some participants discussed feelings of discrimination and racism. Feelings of discrimination were often related to employment experiences, as displayed by the quotes on page 15. Immigrants to Charlottetown commented on the ‘islander’ mentality; long-time residents of the island often refer to immigrants as ‘CFAs’ (Come from Away). The majority of islanders have been raised in a monoculture consisting of mainly White, Anglophone and Christian individuals (Baldacchino et al. 2009). Immigrants may feel that they do not belong in this social network, fostering a sense of perceived discrimination and hindering their socio-cultural integration (Sonn 2002). Some of these experiences and perceptions are similar to the frustrations expressed by the Francophone visible minority participants in Huot et al’s (Huot et al. 2014) study of belonging and migration in London, Ontario.

With respect to community perceptions, the regression analysis revealed that among the entire sample and with immigrant respondents specifically, positive perceptions of neighbourhood schools are associated with improving SoCB. The analysis demonstrated that immigrants place a greater importance on knowing their neighbours on a first name basis and generally trusting people as determinants of an improving SoCB. These perceptions were particularly relevant to immigrant residents of Saskatoon and for those living in Canada for more than 10 years.

To summarize, it is evident (and not surprising given the well-established obstacles that new immigrants face) that from the perspective of a newcomer, the benchmark of having ‘fully arrived’ in Canadian society (and thus feeling a true sense of belonging) is tied to full-time employment (notwithstanding the critical issue of the recognition of foreign credentials) and home ownership. These two factors are undoubtedly more challenging for immigrants when compared to the Canadian-born population. It is quite possible that, in the context of settlement in small to medium sized urban areas, these issues are closely tied to an immigrant’s ability to get to know and trust their neighbours and other members of the community. Further research could determine, for example, if this trust is related to people of a similar ethnic or national background or if the possibilities for integration are greater in smaller urban centres due to smaller immigrant communities.

There a a number of limitations to this research, including the issue of participant social desirability, which may have influenced how immigrant participants responded to both the survey and focus group questions. It is possible that they may have felt pressure to respond positively in order to not be seen as politically incorrect or ungrateful. Further, a larger survey sample size would have allowed a more rigourous examination of the various categories of immigrants, as well as their socio-demographic characteristics. Related to this, the ordinal level data collected in the survey used a limited likert scale that collapsed the responses, disallowing the sensitivity needed to pick-up on the tensions evident in the qualitative data. In retrospect, it would have been useful to analyze if and how the different sizes and ethnic make-up of the three cities shaped the findings in anyway.

## Conclusion

There are several policy recommendations that can be made. First, this research affirms that immigration is critically important to the social and economic well-being of smaller urban areas in Canada. The federal government should continue to work with the provinces to promote immigration to these places by enhancing the Provincial Nominee Program and emphasizing to newcomers and the host communities, the positive possibilities that immigration can bring, as evidenced by many of the findings in this paper. Second, while some progress has been made in this regard, the federal government should make a stronger connection between the skills of potential immigrants and the demands of the labour market, particularly in small-to-medium sized urban areas given the importance of full-time employment to immigrants’ SoCB. Because there are considerable regional economic variations across Canada, the federal government should work with the provinces to identify professions where there is sufficient demand in the labour market, not only at the national level but also at the regional and city levels. The paper found, not surprisingly, that full-time employment leads to better SoCB among immigrants. Tailoring the skills and qualifications of immigrants to specific professions, particularly those where there are shortages, would help to address some of the obstacles that newcomers face.

At the local level, there are a number of public health initiatives that both governments and organizations can undertake to enhance the sense of belonging of newcomers to Canada, and consequently poistively impact their physical and mental well-being. First, it is important that all immigrant service providers (both formal and informal) work together to coordinate services and integrate service delivery to make it easier for immigrants to navigate settlement services and more efficiently find full-time employment. For instance, the Hamilton Immigration Partnership Council (HIPC), formed in 2009, has developed an Immigration Strategy, which aims to achieve this objective. Second, with respect to settlement services, organizations should break-up orientation sessions into multiple visits to make the process for immigrants less overwhelming, while expanding the process to address interests and hobbies, which present opportunities for social inclusion at the local level; this may speed up the process of immigrants better knowing their communities and neighbours. Third, local governments should continue to invest in cultural activities and recreational facilities that meet the needs of the entire population, including recent and longer-term immigrants. This can be achieved, for instance, by creating awareness of current recreation programs through creative and alternative mediums (e.g. ethnic media outlets), while considering immigrant-specific recreation programs. Fourth, local organizations should encourage established residents to reach out and include immigrant neighbours in social activities, whether organized by neighbourhood associations (e.g. block yard sale) or by individuals neighbours themselves (e.g. BBQ). Finally, immigrants themselves should play a leading role in enhancing sense of belonging. Established members of immigrant communities who have lived in a place for an extended period of time are in a position to facilitate the development of a newcomer’s quality of life by offering their assistance with basic needs and providing their knowledge of settlement services and recreation programs.

## Abbreviations

CCHS: Canadian Community Health Survey
QoL: Quality of life
NHS: National household survey
SoCB: Sense of community belonging
